# Molecular profiling of a real-world breast cancer cohort with genetically inferred ancestries reveals actionable tumor biology differences between European ancestry and African ancestry patient populations

**DOI:** 10.1186/s13058-023-01627-2

**Published:** 2023-05-25

**Authors:** Minoru Miyashita, Joshua S. K. Bell, Stephane Wenric, Ezgi Karaesmen, Brooke Rhead, Matthew Kase, Kristiyana Kaneva, Francisco M. De La Vega, Yonglan Zheng, Toshio F. Yoshimatsu, Galina Khramtsova, Fang Liu, Fangyuan Zhao, Frederick M. Howard, Rita Nanda, Nike Beaubier, Kevin P. White, Dezheng Huo, Olufunmilayo I. Olopade

**Affiliations:** 1 The University of Chicago, Chicago, IL USA; 2 Tempus Inc, Chicago, IL USA; 3 Present Address: National University Singapore, Queenstown, Singapore

**Keywords:** Breast cancer, Ancestry, Genomics, Transcriptomics

## Abstract

**Background:**

Endocrine-resistant HR+/HER2- breast cancer (BC) and triple-negative BC (TNBC) are of interest for molecularly informed treatment due to their aggressive natures and limited treatment profiles. Patients of African Ancestry (AA) experience higher rates of TNBC and mortality than European Ancestry (EA) patients, despite lower overall BC incidence. Here, we compare the molecular landscapes of AA and EA patients with HR+/HER2- BC and TNBC in a real-world cohort to promote equity in precision oncology by illuminating the heterogeneity of potentially druggable genomic and transcriptomic pathways.

**Methods:**

De-identified records from patients with TNBC or HR+/HER2- BC in the Tempus Database were randomly selected (*N* = 5000), with most having stage IV disease. Mutations, gene expression, and transcriptional signatures were evaluated from next-generation sequencing data. Genetic ancestry was estimated from DNA-seq. Differences in mutational prevalence, gene expression, and transcriptional signatures between AA and EA were compared. EA patients were used as the reference population for log fold-changes (logFC) in expression.

**Results:**

After applying inclusion criteria, 3433 samples were evaluated (*n* = 623 AA and *n* = 2810 EA). Observed patterns of dysregulated pathways demonstrated significant heterogeneity among the two groups. Notably, *PIK3CA* mutations were significantly lower in AA HR+/HER2- tumors (AA = 34% vs. EA = 42%, *P* < 0.05) and the overall cohort (AA = 28% vs. EA = 37%, *P* = 2.08e−05). Conversely, *KMT2C* mutation was significantly more frequent in AA than EA TNBC (23% vs. 12%, *P* < 0.05) and HR+/HER2- (24% vs. 15%, *P* = 3e−03) tumors. Across all subtypes and stages, over 8000 genes were differentially expressed between the two ancestral groups including *RPL10* (logFC = 2.26*, P* = 1.70e−162), *HSPA1A* (logFC = − 2.73, *P* = 2.43e−49)*, ATRX* (logFC = − 1.93, *P* = 5.89e−83)*,* and *NUTM2F* (logFC = 2.28, *P* = 3.22e−196). Ten differentially expressed gene sets were identified among stage IV HR+/HER2- tumors, of which four were considered relevant to BC treatment and were significantly enriched in EA: ERBB2_UP.V1_UP (*P* = 3.95e−06), LTE2_UP.V1_UP (*P* = 2.90e−05), HALLMARK_FATTY_ACID_METABOLISM (*P* = 0.0073), and HALLMARK_ANDROGEN_RESPONSE (*P* = 0.0074).

**Conclusions:**

We observed significant differences in mutational spectra, gene expression, and relevant transcriptional signatures between patients with genetically determined African and European ancestries, particularly within the HR+/HER2- BC and TNBC subtypes. These findings could guide future development of treatment strategies by providing opportunities for biomarker-informed research and, ultimately, clinical decisions for precision oncology care in diverse populations.

**Supplementary Information:**

The online version contains supplementary material available at 10.1186/s13058-023-01627-2.

## Introduction

Breast cancer (BC) is the most frequently diagnosed cancer in the USA and the second most common cause of cancer-related death in women [1], although there have been significant advances in treatment strategies over the past few decades. In addition to cytotoxic chemotherapy, endocrine therapy, and radiation therapy, in the molecular era, BC patients often receive targeted treatments based on their unique cancer biology in both adjuvant and neoadjuvant settings. For instance, anti-Human Epidermal Growth Factor Receptor 2 (HER2) therapy combined with chemotherapy has become the standard of care in HER2-positive (HER2+) BCs [2, 3]. Hormone receptor-positive (HR+)/HER2- tumors have historically received endocrine therapy and, more recently, CDK4/6 inhibitors as targeted treatments [4, 5]. Some molecularly targeted therapies have also emerged for triple-negative breast cancer (TNBC), such as poly-ADP ribose polymerase inhibitors for *BRCA*-mutated tumors [6, 7] and checkpoint inhibitors for PD-L1+ tumors [9, 10]. Despite these advances, most patients with metastatic BC develop drug resistance or rapidly progress shortly after initiating guideline-informed systemic therapy. Increasingly, genomic testing is used to expand treatment options to overcome de novo or acquired resistance. Patients with HR+/HER2- tumors harboring a *PIK3CA* mutation, for example, experience a prolonged progression-free survival when treated with a phosphoinositide-3-kinase (PI3K) inhibitor in combination with endocrine therapy [8]. However, there is still much room for improvement in endocrine-resistant HR+/HER2- BC and TNBC survival due to their aggressive natures and relatively limited treatment options [9, 10].

TNBC is much less common than the HR+/HER2- subtype in the US population [11], but disproportionately affects patients of African ancestry (AA) compared to those of European ancestry (EA). Moreover, AA patients experience a 40% higher mortality rate than EA patients across all subtypes [11], despite lower incidence rates of BC overall. The complex biologic and social drivers causing these disparities are beginning to be revealed through rigorous studies. While socioeconomic factors like inadequate access to quality care partially contribute to higher mortality, outcome disparities remain even after adjusting for these factors [12]. Several studies point to biological differences in the molecular drivers and evolutionary trajectory of BC based on ancestry. For example, population-based studies have observed higher rates of germline *BRCA1* mutations in AA compared to EA patients [13]. In addition to germline mutations, somatic mutational differences have also been reported. An analysis of tumor sequencing data in the Cancer Genome Atlas conducted by our group revealed a higher prevalence of *TP53* mutations in AA patients and a lower prevalence of alterations in *PIK3CA* [14]. In this same dataset, *TP53* mutations were found to be a positive predictor for recurrence. These data and similar reports demonstrate a significant contribution of genomic differences to the mortality gap between AA and EA patients with BC and point to differential drivers of disease that may be potential therapeutic targets [15].

There is a paucity of molecular and clinical data from underserved and understudied populations, and the few studies using molecular assessments have been underpowered to demonstrate prognostic value in AA patients with BC in the USA [16, 17]. Analyses of comprehensive, large-scale oncology databases are needed to close the knowledge gap and address the unmet clinical need of diverse patient populations. Here, we performed a retrospective analysis of genomic and transcriptomic breast tumor sequencing data from Tempus’ large database and compared results between AA and EA patients. Furthermore, we estimated genetic ancestry employing ancestry-informative markers (AIMs) tailored specifically to our genomic data [18], which can fill gaps in race/ethnicity metadata from electronic health records (EHRs), provide more meaningful biological insights, and increase data availability in under-researched ancestral populations. Combining ancestry estimations, EHR metadata, genomics, and transcriptomics in this real-world cohort may guide the future development of treatment strategies by providing data for biomarker-informed research and precision cancer care.

## Methods

### Cohort selection

A sample of de-identified records from patients with TNBC or HR+/HER2- BC in the Tempus Database was randomly selected (*N* = 5000). A subset of those records was included for analyses after applying relevant inclusion criteria and performing genomic ancestry estimates to identify AA and EA patients (*n* = 3433). Clinical and molecular data were abstracted from patient records as previously described [19]. To be included in the cohort, all patients required results from either the Tempus xT (648-gene targeted panel) or Tempus xE (whole-exome) next-generation sequencing (NGS) assay. Furthermore, only AA or EA patients with known BC subtypes, stages at diagnosis, treatment histories, ages, biopsy sites at the time of testing, and histology (lobular vs. ductal) were selected.

### Classification of patients by genetic ancestry

Although a portion of the patient records contained race and ethnicity metadata obtained from order forms or by abstraction of clinical documents, we opted to estimate global genetic ancestry proportions in the cohort using NGS data. The intent of this approach was to maximize the amount of patient records available for analysis, as race and ethnicity data are sometimes scarce in clinical reports, but also to ensure consistency in comparisons between genetically relevant ancestries and molecular markers. Furthermore, genetic ancestry estimates generally complement self-reported race and/or ethnicity, as we have previously found the two metrics to be concordant [14]. From the representative sample of patient records described above (*N* = 5000), a supervised version of the ADMIXTURE algorithm [20] was applied to a predefined set of AIMs detected in the DNA sequencing data of each patient sample to estimate ancestry proportion likelihoods for five major continental regions: Africa, Americas, East Asia, Europe, and South Asia, as defined in the 1000 Genomes Project (Additional file 1: Table S1) [21]. When a matched normal tissue sample was available (*n* = 3358), the algorithm was run on both tumor and normal NGS data, finding highly concordant ancestry proportions with either specimen for each patient (Africa *r* = 0.999, Americas *r* = 0.9993, East Asia *r* = 0.9997, European *r* = 0.9998, South Asia *r* = 0.9977; Pearson’s r, Additional file 2: Fig. S1).

The ancestry inference algorithm uses 654 and 6711 AIMs overlapping the regions targeted by the Tempus xT and Tempus xE assays, respectively. Considering the selected AIMs and their allele frequencies, ancestry proportion likelihoods for the five aforementioned continental groups were calculated for each patient. Thresholds were then established to determine the genetic ancestry proportions required from each patient to be classified as AA or EA using a combination of admixture proportions reported in the literature for African-American and Hispanic/Latino groups in the USA [22], and by analyzing the ranges of genetic admixture present in patients with available race metadata from clinical records. Genetic ancestry was estimated as AA if likelihoods were > 20% African, < 10% American (Amerindian or Native American), and if the patient reached a total combined African plus European likelihood of > 70%. Conversely, ancestry was estimated as EA if likelihoods were > 80% European and < 10% American. Patient records with genetically determined ancestries other than AA or EA were excluded from subsequent analyses.

### Molecular profiling and comparative analyses

Mutational prevalence from the Tempus xT or xE assay and transcriptional signatures from whole-exome capture RNA-seq were evaluated [23, 24]. Briefly, Tempus xT includes a targeted panel DNA assay evaluating 648 genes, whereas Tempus xE is a whole-exome DNA assay. Among the somatic single-nucleotide variants (SNVs), insertions/deletions (indels), copy number variants (CNVs), and select chromosomal rearrangements identified, variants were annotated as pathogenic or likely pathogenic based on evidence from the Tempus Database including findings from the literature and public databases of association with carcinogenesis or cancer, as previously described [23]. DNA analyses were restricted to the 648 genes included in both platforms, ultimately including 3425 samples from the overall cohort due to quality control (AA *n* = 621, EA *n* = 2804).

The expression of each gene was compared between AA and EA patients with the edgeR software (v3.34.1) in R (v4.1.0) [25], stratified by stage and subtype. Methodology was adapted to account for results from two different versions of the same RNA-seq assay that were included in the cohort. Although both versions were performed based on exome-capture with the same protocol, differences in probe design produced minor batch effects and were accounted for in statistical analyses. Evaluation of differential expression was conducted on samples measured with each assay separately. Then, computed *P* values per gene were combined using the wFisher method [26]. Additionally, log fold-changes (logFC) computed with each assay were combined using a weighted average by sample size for each assay. Results were filtered for genes that had the same logFC directionality between the two assays. There was no significant difference in the proportions of the two different versions of the RNA-seq assay between EA (53% of samples evaluated with version 1, 47% with version 2) and AA samples (49.6% of samples quantified with version 1, 50.4% with version 2).

Gene set enrichment scores were obtained for a series of relevant gene sets using a Python implementation of single-sample gene set enrichment analysis (ssGSEA) based on GSVA v1.46.0 [27]. ssGSEA calculates separate enrichment scores for each pairing of a sample and gene set. Each ssGSEA enrichment score represents the degree to which the genes in a particular gene set are coordinately up- or down-regulated within a sample. For this study, ssGSEA was run using two gene sets: the Molecular Signature Database (MSigDB) hallmark gene set [28], a curated collection of well-defined biological states or processes, and the oncogenic signature gene set [29], which includes signatures of cellular pathways often dysregulated in cancer. These enrichment scores were then compared between AA and EA patients using *limma* [30].

### Statistical analysis

Differences between the mutational prevalence of the genes harboring the highest number of alterations across both AA and EA patients were evaluated by a Pearson's chi-squared test, stratified by subtype. When considering genes beyond the 15 most frequently mutated, *P* values were adjusted for multiple hypothesis testing. The significance of differential gene expression was evaluated by edgeR with Bonferroni correction applied to adjust for multiple testing [25], where a threshold of 0.01 for meta-analysis *P* values was applied resulting in a cutoff of *P* < 1.0e−06. Transcriptional signatures of AA and EA patients were compared and evaluated by *limma* with Bonferroni correction, where a threshold of 0.01 for *P* values was applied resulting in a cutoff of *P* < 4.2e−05. In the portion of the cohort with available race metadata from clinical records, genetic ancestry classifications were compared to metadata using a Pearson's chi-squared test.

## Results

### Cohort overview and genetically determined ancestries

From the initial cohort of patients with BC (*N* = 5000), 623 AA (12.5%) and 2810 EA (56%) patients were identified from NGS-based genetic ancestry inference. When considering the subset of patient records with available metadata of reported race (*n* = 2428), genetically determined ancestries were strongly correlated with the race metadata gathered from clinical settings (97% concordance in AA and 91% in EA, Pearson’s chi-squared test of independence *P* < 2.2e−16; Additional file 3: Table S2). Demographics and clinical characteristics of the final cohort comprising samples with genetically determined ancestries are presented in Table 1. The median ages at diagnosis were similar between AA and EA patients (53.74 and 56.01 years, respectively). The majority of samples sequenced were from cases with metastatic cancer, as stage IV was the most common stage reported in both groups with similar proportions for each ancestry (AA = 83.3% vs. EA = 84.4%, *P* = 0.53; Table 1). HR+/HER2- was the most prevalent subtype in both ancestry groups, although HR+/HER2- diagnoses were relatively more frequent in EA patients (AA = 35.5% vs. EA = 45.6%, *P* = 5.14e−06; Table 1). In contrast, TNBC was relatively more frequent in AA patients (AA = 23.0% vs. EA = 14.2%, *P* = 9.56e−08; Table 1), as expected based on overrepresentation of TNBC in AA populations [11]. Consistent with previous reports, AA patients had higher tumor grades than EA patients across the cohort (AA = 56.8% and EA = 42.6%, *P* = 5.59e−07; Table 1).

**Table 1:** Clinical characteristics of African and European ancestry patients

Characteristic	African ancestry samples (*n* = 623)	European ancestry samples (*n* = 2810)
*Mean (SD), Median age (years)*	54.01 (12.59), 53.74	55.90 (13.37), 56.01
*Stage, n (%)*		
0	0 (0.00)	1 (0.04)
I	11 (1.77)	92 (3.27)
II	48 (7.70)	152 (5.41)
III	45 (7.22)	193 (6.87)
IV	519 (83.31)	2372 (84.41)
*Subtype, n (%)*		
HR+/HER2-	221 (35.47)	1281 (45.59)
Triple-Negative	143 (22.95)	400 (14.23)
Other	259 (41.57)	1129 (40.18)
*Tumor Grade, n (%)*		
Low	163 (26.16)	980 (34.88)
High	354 (56.82)	1198 (42.63)
Unknown	106 (17.01)	632 (22.49)

### Distinct patterns in the mutational landscapes of African and European ancestry breast cancers

**Fig. 1:**
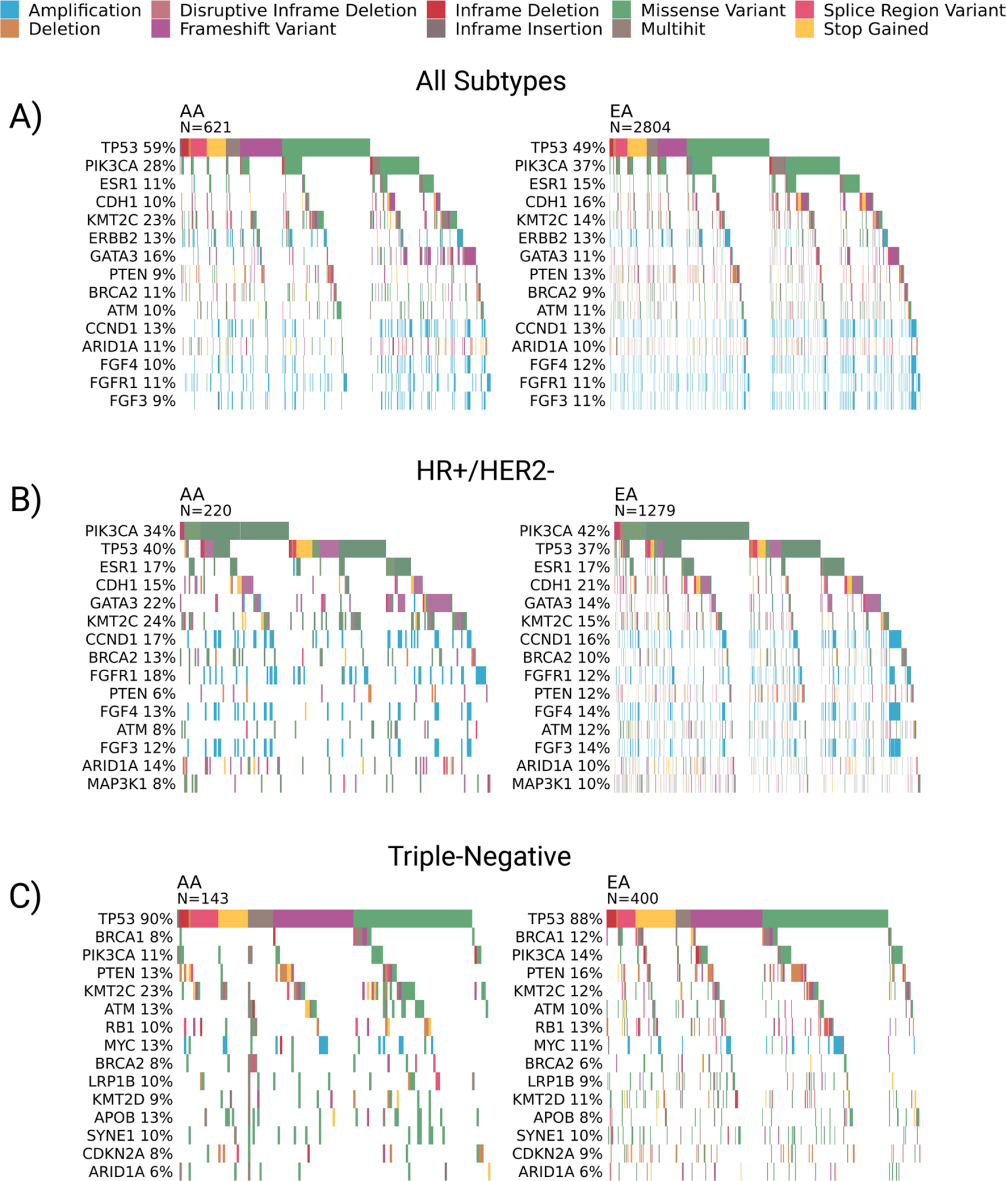
Mutational Landscapes of African and European Ancestry Breast Cancer Patients (AA and EA, respectively). Oncoplots show the top 15 pathogenic mutations in **A** all patients (AA *n* = 621, EA *n* = 2804), **B** patients with HR+/HER2- disease (AA *n* = 220, EA *n* = 1279), and **C** patients with triple-negative breast cancer (TNBC) (AA *n* = 143, EA *n* = 400)

We next evaluated the prevalence of cancer somatic variants (SNVs, indels, and CNVs) classified as pathogenic or likely pathogenic in each ancestral group to identify ancestry-specific differences in tumor DNA. Additional file 4: Table S3 contains the frequency of carriers for alterations in each of the genes compared, the *P* value from a direct comparison between the two ancestry groups, and a *P* value adjusted for multiple hypothesis testing, as well as the most frequent alterations for each ancestry group. In total, frequencies of alterations in 633 genes were compared between AA and EA patients across all subtypes, 625 genes were compared in HR+/HER2- disease, and 612 in TNBC. Considering the top 15 most frequently mutated genes across the entire cohort, *TP53* was the most commonly mutated (AA = 59% vs. EA = 49%; Fig. 1A). When stratifying by subtype, though, the differences in *TP53* frequencies were less pronounced than in the entire cohort (AA = 40% vs. EA = 37% for HR+/HER2- and AA = 90% vs. EA = 88% for TNBC; Fig. 1B and C, respectively).

Among HR+/HER2- disease, the most frequently mutated gene in both ancestral groups was *PIK3CA*, with a significantly higher rate in EA patients (AA = 34% vs. EA = 42%, *P* < *0.05*; Fig. 1B). The rate of *PTEN* mutation was also significantly higher in EA than AA patients (AA = 6% vs. EA = 12%, *P* < 0.05; Fig. 1B). For all other significant differences identified within the HR+/HER2- subtype, AA tumors exhibited higher mutation frequencies, including in *GATA3* (AA = 22% vs. EA = 14%), *KMT2C* (AA = 24% vs. EA = 15%), and *FGFR1* (AA = 18% vs. EA = 12%) (*P* < 0.05 for all; Fig. 1B).

Nonsignificant trends were observed in TNBC patients, such as *BRCA1* mutations being less prevalent in AA compared with EA patients (AA = 8% vs. EA = 12%, *P* = 0.13; Fig. 1C), and *APOB* being mutated at a higher rate (AA = 13% vs. EA = 8%, *P* = 0.09; Fig. 1C). Meanwhile, *KMT2C* mutations were significantly more prevalent in AA than EA patients with TNBC (AA = 23% vs. EA = 12%, *P* < 0.05; Fig. 1C).

### Differential gene expression between African and European ancestry breast cancers

**Fig. 2:**
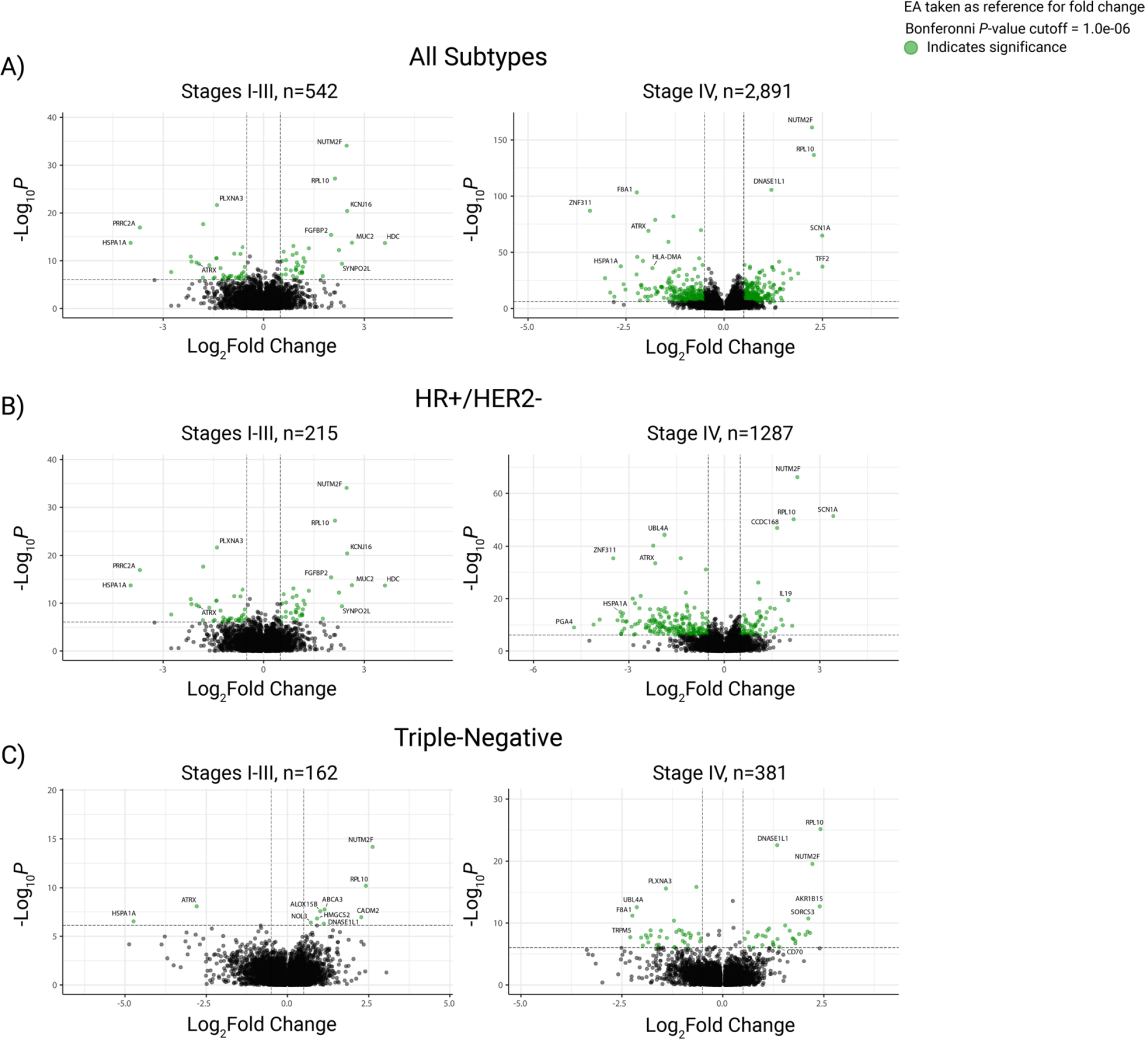
Differential Gene Expression Between Patients of African and European Ancestry (AA and EA, respectively). The volcano plots show all differentially expressed genes between AA and EA patients for **A** all breast cancer subtypes (*n* = 3433), **B** HR+/HER2- samples (*n* = 1502), and **C** triple-negative samples (*n* = 543). Gene sets were plotted by log fold-changes (logFC, *x*-axes) and meta* P* values (*y*-axes). Significance cutoffs were defined for each group, and gene sets with significantly different enrichment between ancestral groups were highlighted in green

Using full-transcriptome RNA-sequencing data, we surveyed gene expression by BC subtype and stage, identifying over 8000 genes with a significantly different expression between the two ancestral groups when considering all subtypes and stages (genes for which *P* < 0.05; Additional file 5: Table S4). The *P* value and logFC (with EA expression as the reference point) for each differentially expressed gene are available in Additional file 5: Table S4. As expected, there were variations in the patterns of differentially expressed genes when stratifying the cohort by subtypes and stages (Fig. 2B and C).

Several genes included in clinical prognostic assays for HR+/HER2- cancer, such as the Oncotype DX Breast Recurrence Score test, were identified as differentially expressed between the two ancestral groups (Fig. 2B, Additional file 5: Table S4). *BAG1* and *BCL2* were expressed significantly higher in AA patients with HR+/HER2- disease (stages I-III and stage IV, respectively), which have both been associated with favorable outcomes [31, 32]. However, many genes related to poor survival were also expressed higher in AA patients compared to EA patients, specifically in the stage IV HR+/HER2- subset, including *BIRC5* [33], *CCNB1* [34], *UBE2C* [35], *CENPA* [36], and *RRM2* [37] (Additional file 5: Table S4). Meanwhile, *SCUBE2*, a gene with high expression linked to favorable outcomes [38], was expressed significantly lower in AA compared to EA patients with stage IV HR+/HER2- tumors (Additional file 5: Table S4).

Although there were less well-established expression-based biomarkers for TNBC, many of the differentially expressed genes identified between ancestral groups could carry prognostic implications in BC or other cancers (Fig. 2C, Additional file 5: Table S4). Interestingly, some of the genes with the strongest logFCs were differentially expressed in most subtypes and stages, such as *HSPA1A* (“All Subtypes, All Stages” logFC = − 2.73, *P* = 2.43e−49) [39], *RPL10* (“All Subtypes, All Stages” logFC = 2.26,* P* = 1.70e−162) [40], *NUTM2F* (“All Subtypes, All Stages” logFC = 2.28, *P* = 3.22e−196), and *ATRX* (“All Subtypes, All Stages” logFC = − 1.93, *P* = 5.89e−83) (Additional file 5: Table S4). Furthermore, genes with implications for therapeutic research, such as those associated with immunotherapy mechanisms, were found to be differentially expressed in TNBCs. For example, *CD70* was significantly higher in AA compared with EA patients among those with stage IV TNBC (logFC = 1.40, *P* = 7.46e−07). Various HLA genes were also differentially expressed across the cohort. *HLA-DRA*, for instance, was significantly lower in AA patients of every subtype and stage combination except for the “All Subtypes, Stage IV” and “HR+/HER2-, Stages I-III” subsets (“All Subtypes, All Stages” logFC = − 1.47, *P* = 9.57E−16; Additional file 5: Table S4).

### Transcriptional signature activation differences between African and European ancestry breast cancers

**Fig. 3:**
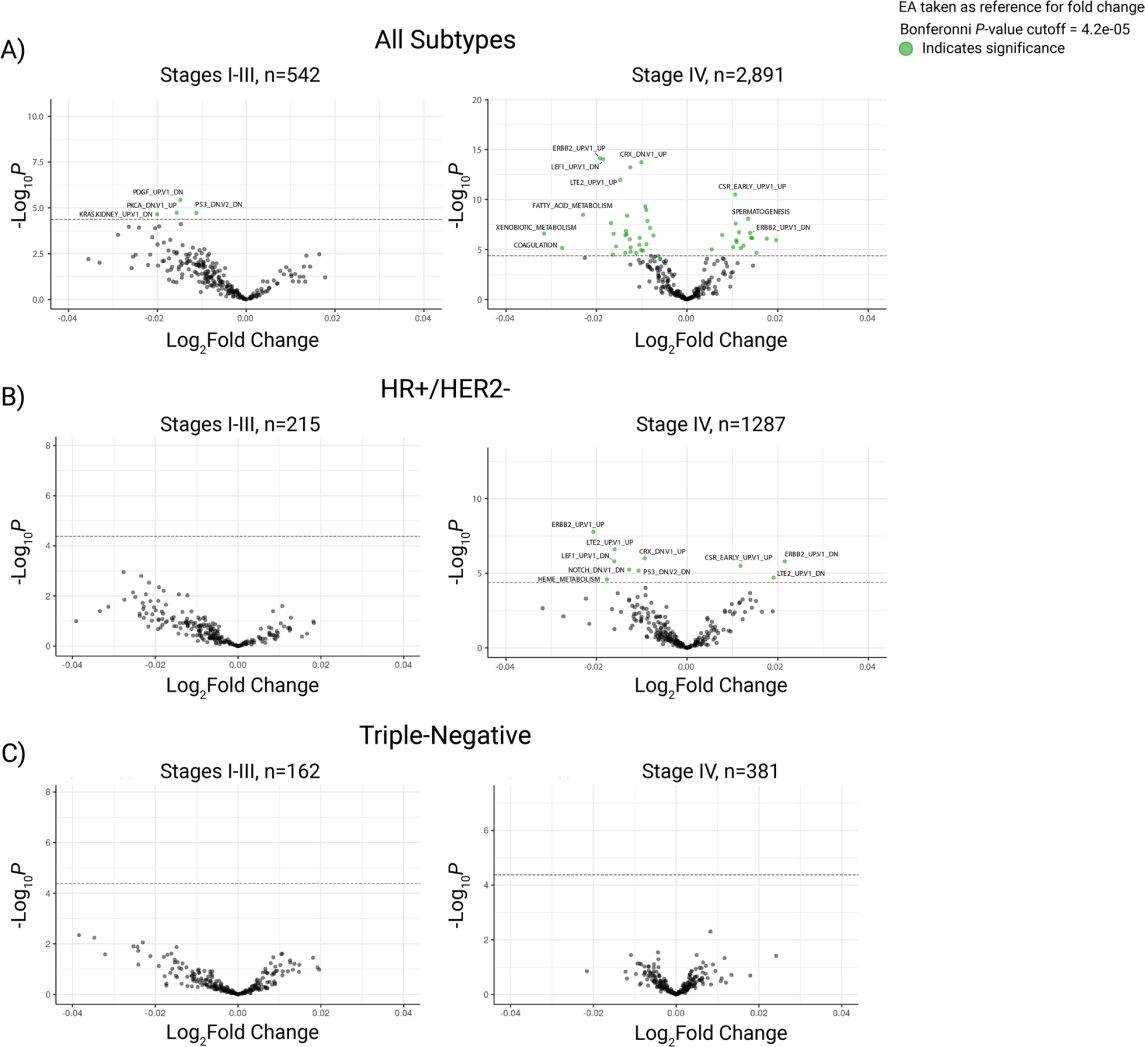
Differential Enrichment of Hallmark and Oncogenic Signature Gene Sets Between African and European Ancestry Patients (AA and EA, respectively). The volcano plots show all differentially expressed gene sets between AA and EA patients for **A** all breast cancer subtypes (*n* = 3433), **B** HR+/HER2- samples (*n* = 1502), and **C** triple-negative samples (*n* = 543). Gene sets were plotted by log fold-changes (logFC, *x*-axes) and *limma P* values (*y*-axes). Significance cutoffs were defined for each group, and gene sets with significantly different enrichment between ancestral groups were highlighted in green

While individual gene expression differences were found between the ancestral groups, simultaneous up- or downregulation of related genes in a pathway can have broader implications for biological processes. To investigate, we measured the differential expression of predefined, biologically relevant gene sets between AA and EA patients using ssGSEA. Figure 3 contains volcano plots displaying a general overview of the differentially expressed gene sets observed. Of the 152 gene sets evaluated, 125 exhibited significantly different enrichment between AA and EA patients across the entire cohort (*n* = 3433, Additional file 5: Table S5) and when stratifying by stage (58 differentially enriched sets by ancestry in stages I-III, *n* = 542, Fig. 3A; 118 sets in stage IV, *n* = 2891, Fig. 3A). Differential enrichments were also observed between ancestral groups in the HR+/HER2- subtype (73 gene sets, *n* = 1502, Additional file 6: Table S5), many of which persisted when only assessing patients with stage IV disease (64 differentially enriched gene sets, *n* = 1287, Fig. 3B) but not in stages I-III (no differentially enriched gene sets, *n* = 215, Fig. 3B). Interestingly, no differences were observed when comparing gene set expression between ancestral groups within the TNBC subpopulation, even when stratifying by stage (Fig. 3C). A full list of gene sets with their associated logFCs and *P* values from ssGSEA is available in Additional file 6: Table S5.

**Fig. 4:**
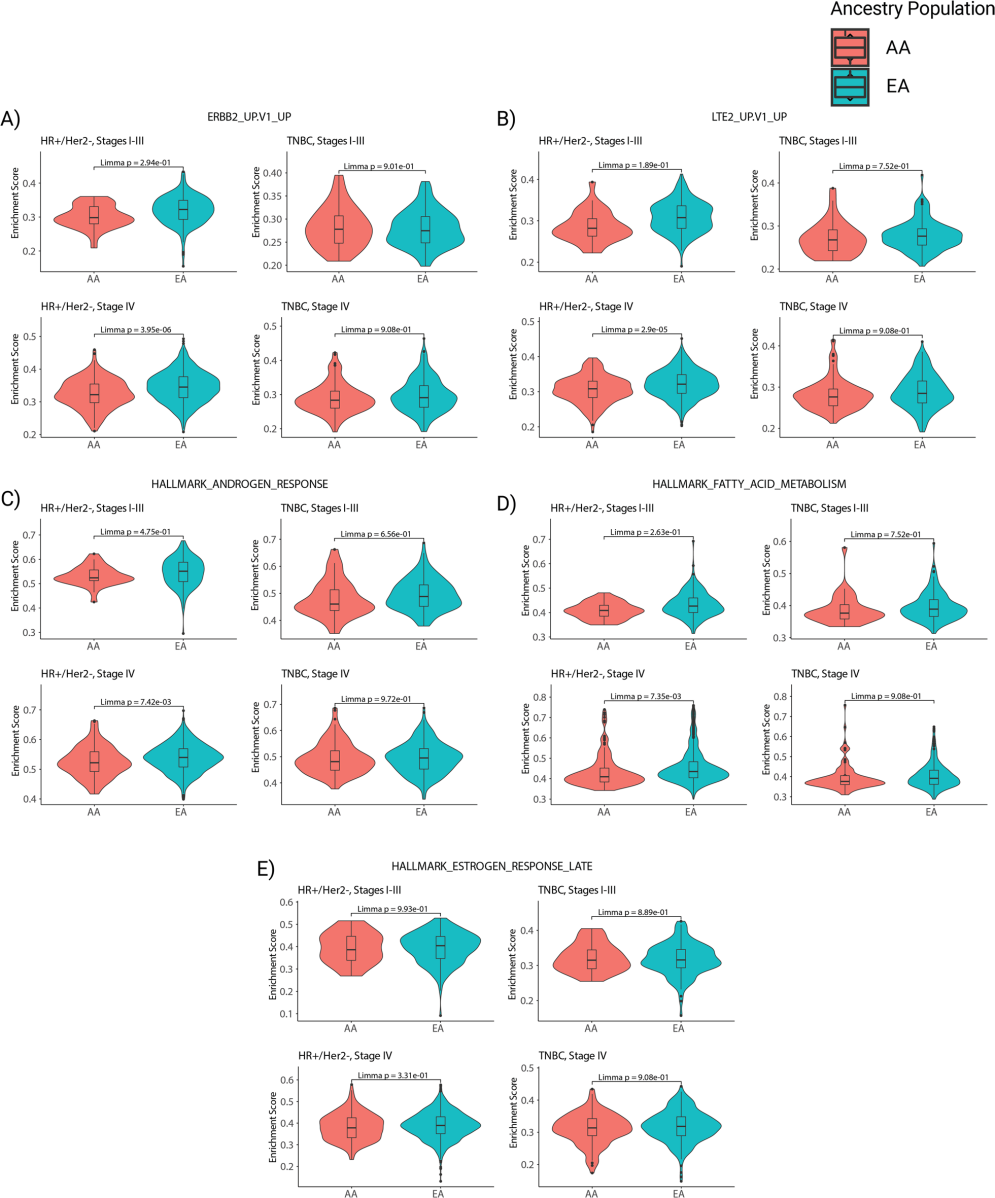
Differential Enrichment of Breast Cancer-Related Gene Sets in African and European Ancestry Patients (AA and EA, respectively). Violin plots depicting five gene sets from the hallmark and oncogenic signature collections that were considered particularly relevant to breast cancer biology, including: **A** ERBB2_UP.V1_UP, **B** LTE2_UP.V1_UP, **C** HALLMARK_ANDROGEN_RESPONSE, **D** HALLMARK_FATTY_ACID_METABOLISM, and **E** HALLMARK_ESTROGEN_RESPONSE_LATE. Only patients with stage IV HR+/HER2- disease exhibited differential enrichment in these gene sets (**A**–**D**), with the exception of the estrogen response pathway (**E**), which had no significantly different enrichment between ancestral groups

From the gene sets in Additional file 6: Table S5, five differentially expressed sets were considered particularly relevant to BC biology: ERBB2_UP.V1_UP, LTE2_UP.V1_UP, HALLMARK_FATTY_ACID_METABOLISM, HALLMARK_ANDROGEN_RESPONSE, and HALLMARK_ESTROGEN_RESPONSE_LATE [28, 41]. Comparisons of ssGSEA results between ancestral groups for each of these BC-related gene sets are presented in Fig. 4, stratified by subtype (HR+/HER2- or TNBC) and stage (I–III or IV). Among stage IV patients with HR+/HER2- disease, ERBB2_UP.V1_UP (Fig. 4A,* P* = 3.95e−06), LTE2_UP.V1_UP (Fig. 4B,* P* = 2.90e−05), HALLMARK_ANDROGEN_RESPONSE (Fig. 4C,* P* = 0.0074), and HALLMARK_FATTY_ACID_METABOLISM (Fig. 4D,* P* = 0.0073) were significantly enriched in EA patients. Again, none of the above gene sets were differentially enriched in any TNBC groups or stage I-III HR+/HER2- patients. Furthermore, there were no significant differences in HALLMARK_ESTROGEN_RESPONSE_LATE enrichment between AA and EA patients within any subtype-stage combination (Fig. 4E).

## Discussion

The results from our large-scale study provide innovative insight by presenting genomic and transcriptomic differences between BC tumors from AA and EA patients, with findings stratified by clinical features in a real-world cohort. The findings indicate the utility of assessing molecular landscapes when conducting both basic research and clinical trials according to the specific ancestries, as well as reveal biologically different mechanisms between AA and EA patients. Importantly, the mechanisms identified carry implications for use of molecularly targeted therapy to broaden early access to clinical trials, including the use of combination therapies in patients with residual disease after neoadjuvant therapy to prevent metastases and late recurrences.

In this cohort, the frequency of *TP53* mutation was higher in AA than EA, which is consistent with previous reports, but there were no significant differences between ancestries in the stratified subtypes [14, 42, 43]. While *TP53* mutations were much more frequent in AA patients with TNBC, our results indicate that the higher frequency of *TP53* mutation in AA tumors from the entire cohort is due to the higher incidence of TNBC in AA. We also observed a significantly lower mutation rate of *PIK3CA* in AA tumors, particularly in patients with HR+/HER2- tumors. Luminal-type tumors are more likely to have *PIK3CA* mutations, providing opportunities for molecularly targeted therapy to improve survival [44]. Several compounds targeting *PIK3CA* have been studied and the US Food and Drug Administration has approved the first PI3K inhibitor alpelisib for advanced luminal BC harboring the *PIK3CA* mutation [8]. While the frequency of *PIK3CA* mutation was lower, any AA patient with identifiable mutations would be expected to benefit from alpelisib, but lack of access to molecular diagnosis could continue to drive racial disparities in outcomes for these patients. This underscores the need to consider each patient as an individual and utilize NGS testing early to capture patients with unique tumor biology and druggable pathways who would benefit from molecularly targeted therapy and/or immunotherapy.

We observed trends suggesting differences in the rates of somatic *BRCA1* and *BRCA2* mutations. Previous studies have reported elevated frequency of *BRCA1* and *BRCA2* germline mutations among AA patients [45–47]; however, the somatic mutation differences by race have not been well documented up until now. While the differences were not significant, we observed a potential racial difference of somatic *BRCA1* and *BRCA2* mutations in both HR+/HER2- and TNBC subtypes. AA patients were more likely to have *BRCA2* mutations within the HR+/HER2- subtype, whereas EA patients were more likely to have *BRCA1* mutations within the TNBC subtype. These findings and previous studies of germline alterations might also have implications for DNA repair-targeted therapies and/or immunotherapies [48].

*KMT2C*, a member of the myeloid/lymphoid or mixed-lineage leukemia family that encodes a histone methyltransferase, is one of the most frequently mutated genes in HR+ BC [49]. The deletion of *KMT2C* has been reported to be associated with resistance to endocrine therapy and worse prognosis [50]. We observed that the frequency of *KMT2C* mutation was significantly higher in AA than EA among TNBC patients (23% vs. 12%) as well as patients with HR+/HER2- tumors (24% vs. 15%), suggesting that the loss of *KMT2C* function might disproportionately affect the survival outcome of AA patients in both subtypes. Previous reports focusing on mutational differences between races also indicated that *GATA3*, which has a critical role in the development of luminal type BC, is mutated in around 10% of both AA and EA patients [42, 43]. In contrast, 22% of AA patients in our cohort had tumors with mutated *GATA3* in HR+/HER2- subtypes, which might contribute to the racial disparity of luminal BC prognosis.

From the full-transcriptome RNA-sequencing data, we identified significantly different expression of over 8000 independent genes between the two ancestral groups. Various genes were exclusively expressed according to subtype (HR+/HER2- or TNBC) or disease stage (stage I-III or stage IV). Here, it is worth mentioning that *RPL10*, which is responsible for DNA replication stress and promoting proliferation and oncogenesis [40], was expressed higher in AA patients throughout almost every subtype and stage. Whereas the higher expression of *RPL10* is reported to be associated with poor prognosis mainly in hematologic malignancy [51, 52], its relation to prognosis in breast malignancy has not been fully investigated. This transcriptomic change in AA patients regardless of subtype or stage might explain the prognostic disparity of AA patients and could be a potential therapeutic target in BC. On the other hand, *HSPA1A*, a member of the heat shock protein 70 family, was significantly lower in AA patients in both HR+/HER2- BC and TNBC. The clinical significance of *HSPA1A* status is unknown in malignant tumors, including BC [53]. *ATRX*, which has a critical role in chromatin remodeling, was also expressed lower in AA patients in both HR+/HER2- BC and TNBC. Previous studies revealed that *ATRX* loss is associated with an increase in cancer aggressiveness [54]. Among a large number of genes with little genomic and clinical annotation, *NUTM2F* was significantly overexpressed in AA patients throughout subtypes and stages. Our comprehensive birds-eye view analysis has identified many potential genes to consider in future basic and clinical research.

When breaking down the enrichment analysis of hallmark gene sets into the subtypes and stages, 125 exhibited significantly different enrichment between AA and EA patients across the entire cohort. No significant differences were observed when comparing gene set expression between ancestral groups within the TNBC subpopulation, even when stratifying by stage. Although the gene sets evaluated here are informative markers of oncogenic pathways, there are other collections of curated gene sets available for analysis, such as the KEGG pathways, and future studies could include these to provide a more thorough investigation of differences between AA and EA patients. Nevertheless, notable differences in many mutations and individual gene expressions between the two ancestral groups in TNBC were demonstrated as an initial assessment.

Meanwhile, among the stage IV HR+/HER2- group, 10 differentially expressed gene sets were identified. As a translation from research to clinic, we revealed four differentially expressed gene sets relevant to BC treatment: ERBB2_UP.V1_UP, LTE2_UP.V1_UP, HALLMARK_FATTY_ACID_METABOLISM, and HALLMARK_ANDROGEN_RESPONSE [28, 41]. In the stage IV HR+/HER2- group, these pathways could contribute to worse prognosis of AA patients and, accordingly, would be worth investigating in further prognostic studies consisting of AA and EA patients.

Our findings could have implications for prognosis, response to therapy, and enrollment of diverse populations in clinical trials and precision oncology studies. If genomic and transcriptomic differences are not considered, applying the results of clinical trials on the studied population (e.g., EA) to another population (e.g., AA) could lead to suboptimal treatment decisions. The molecular distinctions identified here indicate that the efficacy of novel targeted therapy combinations may differ by patient ancestry, and thus highlight an opportunity to optimize neoadjuvant and adjuvant clinical trial design. Additionally, the availability of this data on a population level and for each patient should ultimately result in more clinical trial opportunities including early access to trials for more patients.

Some potential limitations of this study are the broad analyses and heterogeneity of the cohort, as the patients included in the study were highly selected and derived from various institutions and spanned multiple subtypes and stages. Another is the lack of treatment data, as there is likely a mix of treatment-naïve and treatment-refractory patients included in this study, and outcomes data. Nevertheless, in previous studies from our group, we examined NGS data using the Tempus xT assay in patients undergoing neoadjuvant chemotherapy from the Chicago Multiethnic Breast Cancer Cohort and found similar patterns [55]. Furthermore, the patient characteristics were not entirely balanced between AA and EA, considering there were higher-grade tumors and more TNBCs in the AA patient group. Although our large sample size and stratifications by stage and subtype may have ameliorated the impact of differences in patients’ background between the two groups, our analyses did not directly address socioeconomic differences that could play roles in treatment outcomes. The incorporation of those additional factors is beyond the scope of this study and could be included in future studies.

Overall, these data show important differences in BC mutational spectrums, gene expression, and relevant transcriptional pathways between patients with genetically determined African and European ancestries, particularly within the HR+/HER2- BC and TNBC subtypes. To serve diverse populations of patients diagnosed with BC, promote equitable access to clinical trials, and accelerate the development of clinical decision tools for precision care, future studies should focus on geography and genetic ancestry when conducting biomarker-informed, early-phase clinical trials.

## Supplementary Information

**Additional file 1: Table S1:** The predefined set of ancestry-informative markers (AIMs) used to estimate ancestry proportion likelihoods for five major continental regions: Africa, Americas, East Asia, Europe, and South Asia, as defined in the 1000 Genomes Project. The chromosome, position, reference, and alternate allele columns are shown for each of the 654 AIMs.

**Additional file 2: Fig. S1.** Concordance of ancestry proportion likelihood estimates between tumor and normal tissue samples. Overall, samples were highly concordant for estimates of Africa, Americas, East Asia, European, and South Asiaancestries.

**Additional file 3: Table S2:** The subset of patient records with available metadata of reported race, and the number of patient records within each reported race that were inferred as European or African_European_admixed ancestry from next-generation sequencing data.

**Additional file 4: Table S3:** Results from the analysis of cancer somatic variants classified as pathogenic or likely pathogenic in each ancestral group from tumor DNA. Contains the frequency of carriers for alterations in each of the genes compared, the P value from a direct comparison between the two ancestry groups, and a P value adjusted for multiple hypothesis testing, as well as the most frequent alterations for each ancestry group.

**Additional file 5: Table S4:** All genes identified in differential expression analyses by RNA sequencing between African ancestry (AA) and European ancestry (EA) patients. The P value and log fold-change (logFC) with EA expression as the reference point are provided for each differentially expressed gene.

**Additional file 6: Table S5:** Gene sets with significantly different enrichment between AA and EA patients across the entire cohort.

## Data Availability

All relevant data generated during this study are included in this published article and its supplementary information files. Raw data are not available due to proprietary restrictions.

## Abbreviations

BC: Breast cancer
TNBC: Triple-negative breast cancer
AA: African ancestry
EA: European ancestry
LogFC: Log fold-change
HER2: Human epidermal growth factor receptor-2
HR: Hormone receptor
AIM: Ancestry-informative marker
EHR: Electronic health record
NGS: Next-generation sequencing
ssGSEA: Single-sample gene set enrichment analysis
