# Supplementary figures and images for: Molecular profiling of a real-world breast cancer cohort with genetically inferred ancestries reveals actionable tumor biology differences between European ancestry and African ancestry patient populations
Source: Breast Cancer Res. 2023 May 25;25:58. doi: 10.1186/s13058-023-01627-2 (PMC10210411; doi:10.1186/s13058-023-01627-2)

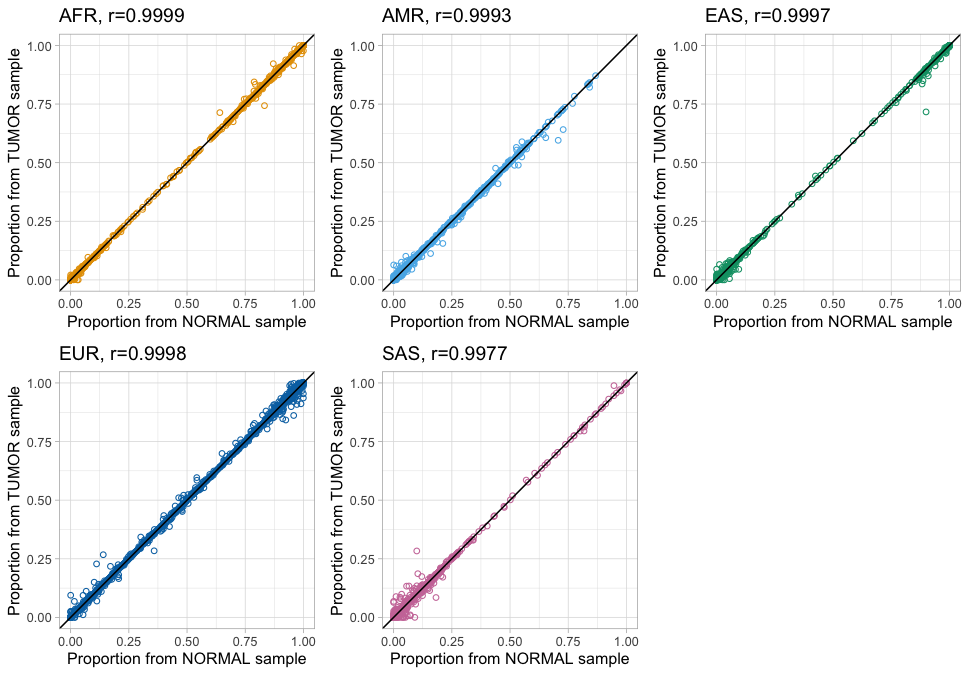

Supplement: Supplementary file 2 — Additional file 2: Fig. S1. Concordance of ancestry proportion likelihood estimates between tumor and normal tissue samples. Overall, samples were highly concordant for estimates of Africa, Americas, East Asia, European, and South Asiaancestries. [file 13058_2023_1627_MOESM2_ESM.png]
